# Development of a Digital Multimodal Pain Intervention (CHRONIC): Protocol for a Randomized Controlled Trial Study

**DOI:** 10.2196/84437

**Published:** 2026-05-26

**Authors:** Gentian Bunjaku, Lena Fellbaum, Aaron Laufhütte, Tanja Zimmermann

**Affiliations:** 1 Department of Psychosomatic Medicine and Psychotherapy, Medical School Hannover, Carl-Neuberg-Str. 1, Hannover, 30625, Germany, 49 511 532 6347; 2 See Acknowledgments

**Keywords:** chronic pain, telemedicine, cognitive behavioral therapy, acceptance and commitment therapy, mindfulness, self-help program, digital health application, DiGA

## Abstract

**Background:**

Chronic pain affects approximately 12 million individuals in Germany and significantly impairs quality of life. Although multimodal treatment approaches—combining physical, psychological, and behavioral strategies—are considered the gold standard, access to specialized multidisciplinary pain treatment, including physiotherapy and psychological interventions, is often hampered by long waiting times, which highlights the need for scalable, low-threshold treatment options. Digital health interventions, particularly those grounded in established therapeutic models, offer a promising solution to bridge this treatment gap. The German Digital Health Care Act has opened pathways for reimbursable digital applications, such as CHRONIC, which is grounded in acceptance and commitment therapy as a third-wave cognitive behavioral therapy framework and integrates selected cognitive behavioral techniques, mindfulness-based strategies, and physiotherapy into a structured web-based program.

**Objective:**

This study protocol outlines a randomized controlled trial (RCT) aimed at evaluating the effectiveness of CHRONIC, a 12-week web-based, multimodal self-help program for individuals with chronic pain, with a focus on reducing pain intensity, reducing pain-related interference, and improving quality of life.

**Methods:**

This is a monocentric, 3-arm RCT with assessments up to 6 months after baseline. A total of 200 participants with chronic nonmalignant pain will be randomized into one of three groups: (1) intervention with individualized psychological feedback, (2) intervention with standardized computer-generated feedback, and (3) treatment-as-usual waitlist control. Weekly modules in the intervention arms include psychotherapeutic video sessions, interactive acceptance and commitment therapy–based and cognitive behavioral therapy–based exercises, mindfulness practices, and physiotherapeutic training. Outcomes will be assessed via validated self-report instruments: West Haven-Yale Multidimensional Pain Inventory for pain intensity and interference, Assessment of Quality of Life—8 Dimensions for quality of life, German version of Chronic Pain Acceptance Questionnaire for pain acceptance, Patient Health Questionnaire-9, German version for depression, and Generalized Anxiety Disorder 7-item Scale for anxiety. Data will be analyzed using mixed models for repeated measures based on the matched ~6-month assessment. Missing data will be addressed using multiple imputation.

**Results:**

Recruitment started in January 2026 and is projected to end in July 2026. As of January 22, 2026, 18 participants had been enrolled. Primary end point data collection is expected to be completed in January 2027, with primary analyses and dissemination planned for 2027.

**Conclusions:**

This study protocol describes an RCT designed to evaluate the clinical utility of CHRONIC as a scalable, low-threshold intervention for chronic pain. CHRONIC aims to address the multifactorial nature of chronic pain by integrating psychotherapeutic, physiotherapeutic, and mindfulness-based strategies into a single digital program. Future studies will be required to determine how this multimodal format compares to unimodal interventions. The findings aim to inform future digital health developments and contribute to the implementation of biopsychosocial care models in routine practice.

## Introduction

### Background

Approximately 12 million individuals in Germany are affected by chronic pain [1], representing around 14% of the national population. Alongside physiotherapy and medication, psychotherapeutic interventions have proven effective in treating chronic pain [2]. However, extensive waiting times of several months before patients receive access to specialized multidisciplinary pain services represent a major barrier to adequate pain management and contribute to delayed treatment initiation and symptom chronification [3], highlighting the urgent need for scalable, cost-effective treatment options. To address this gap, the German government implemented the Digital Healthcare Act (Digitale-Versorgung-Gesetz) on December 9, 2019, enabling physicians and psychotherapists to prescribe reimbursable digital health applications. Parallel to these developments, international research has demonstrated that digital psychological interventions for chronic pain, and particularly those based on acceptance and commitment therapy (ACT) and cognitive behavioral therapy (CBT), can effectively reduce pain interference and distress compared with waitlist control conditions [4-6]. For example, the German online ACT program ACTonPain showed medium effects on pain-related functioning and pain acceptance in individuals with long-standing pain symptoms, with improvements sustained at follow-up. Importantly, ACTonPain compared guided and unguided ACT delivery and found significant improvements in both intervention arms relative to waitlist control, while guided ACT demonstrated stronger effects and lower attrition rates, highlighting the potential clinical value of blended or therapist-supported components in digital pain treatment [4]. However, most existing digital interventions primarily reflect unimodal treatment formats focusing on psychological or physical mechanisms in isolation. Given the multidimensional nature of chronic pain and the limitations of unimodal approaches, there is a need for integrated, multimodal digital interventions that combine psychotherapeutic, behavioral, and physical activation strategies within a single framework. The rationale for developing a multimodal digital intervention is further supported by research demonstrating that chronic pain outcomes improve most when biological, psychological, and behavioral mechanisms are targeted simultaneously. Meta-analytic findings show that multimodal pain rehabilitation programs outperform unimodal approaches in terms of pain interference, disability, and emotional functioning [7,8]. Digital delivery formats are increasingly used to operationalize such biopsychosocial principles: blended or hybrid pain interventions combining psychological content, physical exercise components, and self-management strategies have reported clinically meaningful improvements in pain intensity, mobility, and quality of life [9,10]. These results suggest that integrating physiotherapeutic and psychological principles within a single digital platform may strengthen treatment effects by addressing multiple pain mechanisms concurrently rather than sequentially. The web-based application CHRONIC is designed to address this care gap for individuals with chronic pain. CHRONIC is grounded in ACT [11] as a third-wave cognitive-behavioral approach and integrates selected traditional CBT techniques (eg, cognitive restructuring and behavioral activation), mindfulness-based strategies [12], and physiotherapeutic exercises into a coherent, multimodal program. The goal is to offer a comprehensive educational and therapeutic program that equips users to manage their pain condition effectively. Given the multidimensional nature of chronic pain, a multimodal treatment approach—integrating biological, psychological, and social elements—is considered necessary [13]. Skelly et al [14] found that unimodal nonpharmacological interventions for chronic pain—such as isolated exercise or psychological therapies—often produce only modest and short-term effects. Long-term benefits were rare, and methodological heterogeneity limited the strength of evidence. The authors conclude that single-modality treatments fail to address the biopsychosocial complexity of chronic pain, highlighting the need for integrated, multimodal approaches.

### Identification and Treatment of Chronic Pain

Pain is defined by the International Association for the Study of Pain as a persistent sensation following the expected healing time of an acute injury [15]. Due to variability in recovery durations depending on the nature of the injury, clinical practice commonly applies a fixed time threshold to define chronic pain. For instance, low back pain is classified as chronic when persisting beyond 6 months, whereas postherpetic neuralgia may be considered chronic after only 3 months [16].

The *International Classification of Diseases—11th revision* has improved the classification of chronic pain by introducing clinically relevant categories and specific diagnostic criteria [17]. Chronic pain is now divided into seven distinct categories: (1) chronic primary pain, (2) chronic cancer-related pain, (3) chronic postsurgical and posttraumatic pain, (4) chronic neuropathic pain, (5) chronic headache and orofacial pain, (6) chronic visceral pain, and (7) chronic musculoskeletal pain. All categories share the criterion of persistent or recurrent pain for a minimum duration of 3 months, with optional specifiers addressing psychosocial factors and severity. The latter is assessed through pain intensity, interference, and functional limitations. Each category also emphasizes the significant impairment in quality of life and the necessity to exclude other potential etiologies before diagnosis [18].

A review by Hylands-White et al [13] outlined the diverse range of chronic pain treatments. Pharmacologic approaches such as analgesics may offer short-term relief but often show limited effectiveness in chronic nonmalignant pain. Even opioids typically reduce pain by only 30%, with potential for tolerance, side effects, and reduced long-term benefits. Nonsteroidal anti-inflammatory drugs are more effective in some cases but carry risks such as gastrointestinal bleeding and cardiovascular events, particularly with long-term or high-dose use. Topical analgesics offer lower systemic risk, though concerns regarding dependency persist—especially for opioid-based agents [19]. Adjuvant medications, including anxiolytics, hypnotics, antidepressants, and anticonvulsants (often used off-label), are also possible approaches. Nonpharmacological treatments include spinal cord stimulation, deep brain stimulation, repetitive transcranial magnetic stimulation, transcranial direct current stimulation, counter-irritation, transcutaneous or percutaneous electrical nerve stimulation, topical capsaicin, and thermotherapy or cryotherapy.

However, the inherently multidimensional nature of chronic pain necessitates a biopsychosocial framework that integrates biological, psychological, and social interventions. Physiotherapy contributes by maintaining or restoring physical function, while psychotherapeutic techniques from CBT and ACT assist patients in coping with chronic pain, implementing stress management strategies, and achieving emotional stabilization [13]. Digital interventions in chronic pain management show significant promise but require further rigorous investigation to establish their evidence base and long-term effectiveness [20,21]. Currently, in Germany, just one digital health application is officially listed and reimbursable for chronic pain treatment: “HelloBetter Chronische Schmerzen,” an interactive online program based on ACT techniques, including psychoeducation, videos, and audio [22]. This program primarily addresses the psychotherapeutic dimension of chronic pain but does not include physiotherapeutic components. Therefore, the inclusion of applications such as CHRONIC, which incorporates both physical activation and psychological guidance, would represent a valuable extension of current digital care offerings within a truly biopsychosocial framework.

### The Future Study

The aim of the future study is to evaluate the effectiveness of the digital intervention CHRONIC for individuals with chronic pain. In light of the increasing availability of web-based programs targeting chronic pain, CHRONIC adopts a multimodal approach that distinguishes itself from existing interventions by integrating multiple evidence-based components rather than relying solely on a singular therapeutic framework, thus providing a truly biopsychosocial framework. To enhance compliance and user engagement, the program incorporates a video-based therapist who guides participants through interactive modules, thereby fostering a sense of therapeutic alliance. In addition, personalized feedback is provided to further support adherence and individualization of the intervention process.

### Study Objectives

The following research questions and objectives are to be examined in the context of evaluating the 12-week web-based self-help program CHRONIC for individuals with chronic pain:

1. Reduction of pain intensity: Does participation in CHRONIC lead to a reduction in experienced pain intensity compared to a treatment-as-usual (TAU) group at postintervention (week 12) and the primary end point (6 mo after baseline)?
2. Reduction of pain-related impairment: Does CHRONIC reduce the degree to which chronic pain interferes with daily life and functioning compared to TAU at postintervention (week 12) and the primary end point (6 mo after)?
3. Improvement in quality of life: Does participation in CHRONIC enhance participants’ quality of life compared to TAU at postintervention (week 12) and the primary end point (6 mo after)?
4. Increase in pain acceptance: Does CHRONIC support participants in developing a higher level of acceptance regarding their chronic pain compared to TAU at postintervention (week 12) and the primary end point (6 mo after)?
5. Comparison of feedback formats: Does template-based, computer-generated feedback offer comparable benefits to individualized therapist feedback within a web-based self-help setting? Specifically, do participants receiving standardized feedback (experimental group with computer-generated feedback [EGcomp]) differ significantly from those receiving personalized feedback (experimental group with individualized psychological feedback [EGpsych]) regarding primary outcomes?

The overall aim is to evaluate the effectiveness of CHRONIC as a low-threshold, scalable intervention for individuals with chronic pain and to explore the potential of automated feedback systems within digital health interventions.

## Methods

### Participants

The study population consists of individuals with chronic pain. Inclusion criteria are as follows: (1) chronic nonmalignant pain defined as pain in one or more anatomical regions persisting for more than 3 months, present on most days of the week, and associated with significant emotional distress or functional disability, (2) a minimum age of 18 years, (3) sufficient knowledge of German, and (4) regular access to an internet-enabled device (computer or tablet). Exclusion criteria include acute suicidal ideation, uncorrected hearing impairments, significant language barriers, and current participation in psychotherapy. Current participation in structured psychotherapeutic treatment for chronic pain is an exclusion criterion to avoid overlap with the psychotherapeutic components of CHRONIC. Concurrent physiotherapy and routine medical care are allowed because the physiotherapeutic component of CHRONIC is intended as an adjunct to routine physical care but should be recorded and considered in sensitivity analyses.

### Study Design

**Figure 1.**
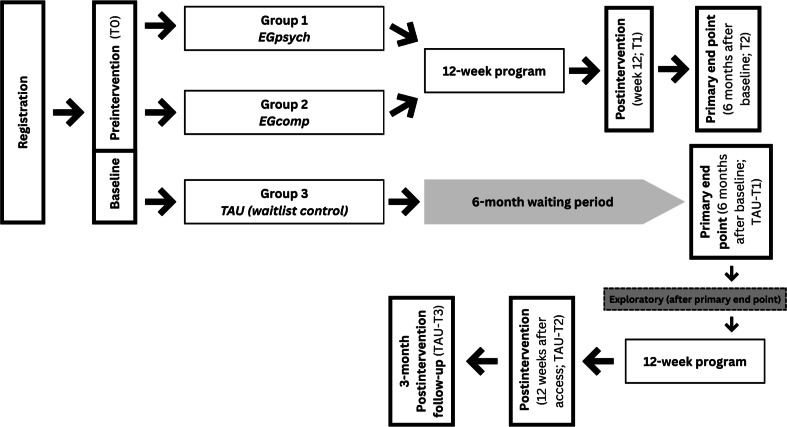
Study design.

This is an interventional, randomized controlled trial (RCT) with 3 parallel arms, including a nonblinded TAU group. The 2 experimental groups (EGpsych and EGcomp) complete 3 assessments: baseline prior to intervention start (T0), postintervention at week 12 (T1), and a 3-month postintervention follow-up, which represents the primary end point (6 mo after baseline, T2). In contrast, the TAU group follows an extended assessment schedule due to the 6-month waiting period: TAU participants complete a baseline measurement at study entry (T0), a second assessment following the 6-month waiting period and immediately before accessing the intervention (TAU-T1), a postintervention assessment after completing the 12-week program (TAU-T2), and a 3-month follow-up (TAU-T3). The 2 experimental groups receive access to the digital self-help program CHRONIC. Participants in the TAU group will receive access to the CHRONIC program with standardized computer-generated feedback (EGcomp version) after completing the 6-month waiting period. The 6-month waiting period for the TAU group was selected for 3 reasons. First, it reflects real-world access barriers in specialized pain services in Germany, where waiting times of 4 to 6 months are frequently reported for multidisciplinary pain treatment programs and specialist behavioral interventions, making this duration clinically realistic rather than arbitrary [3,23]. Second, extending the waiting period increases the likelihood of detecting natural symptom progression and pain chronification effects, allowing a meaningful comparison with the intervention arms and preventing artificially low variance caused by very short control periods. Third, choosing a 6-month window enables methodological alignment with the intervention design: since participants in the experimental arms undergo a 12-week treatment followed by a 3-month follow-up, the 6-month TAU period ensures comparable temporal exposure before receiving access to the program. The groups differ in the type of feedback received after each module: participants in the EGpsych receive individualized feedback written by the study team, whereas participants in the EGcomp receive standardized, template-based, computer-generated feedback. The study design is illustrated in Figure 1.

### Ethical Considerations

Ethical approval was granted by the Ethics Committee of the Hannover Medical School (approval ID 11563_BO_S_2024), and the trial was prospectively registered in the German Clinical Trials Register (ID DRKS00036901) on May 19, 2025.

All participants were informed about the purpose and procedures of the study, the voluntary nature of participation, and their right to withdraw at any time without disadvantage. Informed consent was obtained from all participants prior to their participation in the study.

The authors confirm that all collected data were handled confidentially and processed in compliance with applicable data protection and privacy regulations. No personally identifiable information is disclosed in this manuscript.

### Sample Size

An a priori power analysis was conducted in G*Power (version 3.1.9.7; Erdfelder, Faul, and Buchner) to estimate the sample size required to detect a small-to-medium longitudinal intervention effect in a group comparison. Consistent with common planning approaches for repeated-measures trials, the calculation targeted the group × time interaction under conservative assumptions and serves as an approximation for the primary longitudinal analyses. We assumed an effect size of *f*=0.20, *α*=.025 and statistical power=.95. The correlation among repeated measures was set to *r*=0.50 and the nonsphericity correction to *ε*=0.75 to account for plausible deviations from perfect sphericity. Under these assumptions, G*Power yielded a minimum required sample size of 114. To account for dropout, a target sample size of 200 was prespecified. This inflation reflects the increased risk of dropout in the TAU control group due to the 6-month waiting period and the typical loss to follow-up in digital health trials. The larger sample ensures robustness against assumption violations and maintains adequate power.

### Procedure and Recruitment

Participants are recruited via digital platforms (eg, online forums and patient advocacy groups) as well as through direct cooperation with health care providers (eg, pain clinics, physiotherapy practices, and orthopedic clinics). Interested individuals are provided with detailed study information through an online portal, including an introductory video explaining the intervention. Support is available via a dedicated project email address. Upon registration and consent, participants undergo an onboarding process to assess eligibility and complete the baseline measurement (T0). All assessments are administered digitally through the intervention platform. Participants are pseudonymized using computer-generated codes. The link between codes and participant information is stored separately in an encrypted file accessible only to the study team. This file allows reidentification solely for data deletion upon participant request, ensuring compliance with data protection standards.

### CHRONIC Web Application

CHRONIC is a digital, web-based intervention grounded in a combination of ACT [11] and CBT. The program is further enhanced by the integration of mindfulness-based strategies and physiotherapeutic components, drawing on practical experience in the treatment of patients with chronic pain. Over the course of a 12-week self-help program, users are guided to develop a deeper understanding of their pain condition and of themselves, foster pain acceptance, improve emotional regulation and stress management, and define concrete personal goals for future behavior and life planning. Core components include strengthening personal responsibility and building internal and external resources. From a physiotherapeutic perspective, the intervention promotes physical functionality through posture training, a full-body exercise program (adaptable in intensity), and practical knowledge for maintaining safe gait and stance to support current and future mobility. Mindfulness exercises are interspersed between modules to sharpen external awareness and enhance attentional control. Table 1 provides an overview of the program modules and their respective contents. To ensure clinical relevance and theoretical coherence, the content and sequencing of the CHRONIC modules were derived from a combination of empirically supported treatment principles and clinical experience in treating individuals with chronic pain. The psychotherapeutic modules are grounded in core components of ACT, whose effectiveness in digital self-help formats for chronic pain has been demonstrated, for example, in the evaluated digital health application “HelloBetter Chronischer Schmerz.” Complementing this, the physiotherapeutic components were selected based on real-world clinical demands frequently observed in outpatient settings: physical deconditioning, insecure movement patterns, and fear of movement. To address these, CHRONIC includes functional exercises (adaptable in intensity), postural guidance, and educational input on safe movement execution, aiming to promote mobility confidence and bodily self-efficacy. Mindfulness-based exercises interwoven throughout the program serve to enhance interoceptive and attentional awareness, supporting both psychological flexibility and functional engagement. Overall, the selection of modules reflects a biopsychosocial rationale: each session builds on previous content and targets a distinct yet interrelated aspect of pain coping. This structure allows participants to develop adaptive cognitive, emotional, behavioral, and physical strategies in a coherent, cumulative manner over the course of the 12-week program.

**Table 1.** Overview of the CHRONIC program modules.

Module	Description of content
1. Pain and now?	Psychoeducation on the topic of chronic pain, including an overview of the program and introduction of the assigned therapist.
2. Becoming fully awake	Participants are introduced to the concept of mindfulness, which involves observing the present moment without judgment. This session focuses on cultivating the ability to consciously direct attention to the here and now.
3. Understanding pain	Accepting the current state of one’s condition is often perceived as counterintuitive or even unimaginable. This session explores why acceptance can nonetheless lead to long-term improvement in symptoms and overall functioning.
4. Do you feel that too?	Emotions are a constant part of human experience. While some are perceived as pleasant and others as distressing, this session addresses strategies for managing the full range of emotional experiences in an adaptive manner.
5. Don’t stress me out	Stress is a common experience; yet in the context of chronic pain, it can become particularly overwhelming. This session provides techniques for more effective stress regulation.
6. Bird’s-eye view	Defusion refers to a mental state in which thoughts and emotions are observed without becoming entangled in them. Participants learn how to develop a more distanced and less reactive stance toward internal experiences.
7. What matters to me	Personal beliefs significantly influence both cognition and behavior. Conversely, engaging in actions aligned with one’s core values can help reinforce a meaningful and coherent sense of self. This session explores this bidirectional relationship.
8. Commitment of the self	Living a meaningful life requires ongoing commitment. This session introduces the concept of value-based goal setting and encourages participants to formulate personal commitments that support long-term well-being.
9. In the here and now	People often dwell on the past or worry about the future. This session highlights the benefits of present-focused awareness and offers practical guidance for anchoring attention in the here and now.
10. The creature of habit	Changing entrenched behavior patterns is inherently difficult. In this session, participants reflect on habitual behaviors and explore strategies for initiating and maintaining adaptive behavioral change.
11. Reinforcement from within	This session centers on personal resources—both internal and external—that contribute to resilience and psychological flexibility. Participants are encouraged to identify, sustain, and further develop these resources.
12. …and last but not least	The final session acknowledges the participants’ engagement throughout the program and emphasizes the importance of continuing to invest in one’s health and well-being beyond the structured intervention.
Physiotherapy module 1	Introduction to physiotherapeutic principles: education on ergonomic posture, everyday movement patterns, and alignment correction to reduce pain load during daily activities.
Physiotherapy module 2	Guided whole-body exercise routine designed to improve strength, stability, and mobility; intensity levels adjustable by participants based on functional capacity and pain level.
Physiotherapy module 3	Movement retraining for walking and standing; focus on biomechanical efficiency and safety.

The program is structured so that participants complete one psychotherapeutic unit per week, followed directly by either a physiotherapy or mindfulness-based unit. Each weekly session is designed to take approximately 50 minutes, with the intention that users apply the learned content between sessions in their everyday lives. Users can revisit all materials as often as they wish during the 12-week access period. The psychotherapeutic sessions are delivered via video recordings of a licensed psychological psychotherapist, who explains the core content of each session and poses interactive questions during the modules. Videos pause automatically when user input is required, and corresponding exercises are displayed on screen. Upon completion of an exercise, marked by the user, the video continues. At the end of each session, users can download their completed exercises or obtain blank worksheets for printing and future reuse.

Modules are time-locked to ensure standardized intervention exposure because the intervention is designed to run over a fixed 12-week period. Each new module becomes available exactly 7 days after the previous module has been released. Participants cannot access future modules ahead of schedule, and early progression or skipping forward is technically prevented. The program cannot be paused. All participants have a fixed consecutive 12-week access period, after which access to the intervention ends automatically. If participants choose not to complete a module in the week it becomes available, the next module is still released on schedule 7 days later. This allows participants to complete multiple outstanding modules in a shorter interval later on, reducing the time gaps between sessions while still operating within the fixed 12-week intervention window. All completion times and access patterns are recorded through backend timestamps and will be evaluated in adherence. To support structured engagement and continuity in self-monitoring, brief intersession assessments are implemented within the platform workflow. Before participants can begin the next module, they are required to complete a short check-in assessment referring to the preceding week, based on subscales derived from the West Haven-Yale Multidimensional Pain Inventory (WHYMPI; part 1 “Pain experience statements” and part 3 “Patient activity”). These assessments are integrated directly into the platform interface and are automatically presented at the start of each new session. The check-ins occur once per week, require approximately 2 minutes to complete, and refer to the participant’s experiences during the previous 7 days. Modules cannot be accessed until the corresponding check-in is completed, ensuring a consistent measurement structure across participants. There is no fixed time of day for completion since the brief assessment appears immediately when the new module is opened and remains available until submitted. These data are visualized in real time on the user’s personal dashboard as a line graph based on the summed scores to support self-monitoring. After each module, participants in the experimental groups receive motivational feedback—either individualized or computer-generated, depending on group assignment—delivered via the app and email shortly after session completion. Feedback is only triggered following the completion of a module. The feedback procedure differs between the 2 intervention conditions. In the EGpsych arm, feedback is written individually by psychologists from the study team. All potential feedback providers hold a university degree in psychology and have additional clinical experience in working with individuals with chronic pain. Feedback integrates participant-specific information derived from the weekly self-assessments. Messages are delivered asynchronously in written text format via the platform interface. In contrast, participants in the EGcomp arm receive standardized feedback messages prewritten by psychologists from the study team based on a fixed template system. These messages include the same structural components as in EGpsych (completion acknowledgment, motivational reinforcement, psychoeducational element, and goal reminder), but they do not integrate individual self-assessment data or module-specific personal content. The feedback is identical across all EGcomp participants and does not vary based on adherence patterns or timing. This standardized structure ensures consistency in length, tone, and delivery frequency across participants while maintaining methodological separation from tailored guidance. In both intervention arms, feedback messages are delivered asynchronously via the platform and email notification within 24 hours following module completion, and participants cannot respond to feedback in either condition. The feedback texts contain approximately 100 to 150 words per module. If participants complete multiple modules within a single day (catch-up use), EGpsych feedback is not provided separately for each module. Instead, one feedback message is provided referencing the last completed module and comparing the most recent assessment with the prior assessment, thereby reflecting the latest available change pattern rather than generating multiple module-specific feedback messages. Such accelerated completion patterns and potential overlaps in assessment windows are captured via timestamps and will be considered in adherence metrics and per-protocol (PP) definitions.

### Measurements

To measure the study objectives, the following outcomes will be captured: (1) *pain intensity*, (2) *pain impairment*, (3) *quality of life*, (4) *physical and emotional functionality*, (5) *pain acceptance*, and (6) *anxiety and depressive symptoms*. A list of measurement instruments is presented in Table 2.

**Table 2.** Measurement instruments^a^.

Questionnaire	Pre	Post	Follow-up
Basic data			
Demographics (age, sex, education, etc)	✓	—^b^	—
Primary outcomes			
Pain intensity and pain impairment (WHYMPI^c^)	✓	✓	✓
Quality of life (AQoL-8D^d^)	✓	✓	✓
Secondary outcomes			
Physical functionality (WHYMPI subscales)	✓	✓	✓
Emotional functionality (WHYMPI subscales)	✓	✓	✓
Pain acceptance (CPAQ-D^e^)	✓	✓	✓
Anxiety (GAD-7^f^)	✓	✓	✓
Depression (PHQ-9^g^)	✓	✓	✓

a Treatment as usual (TAU) includes additional assessments due to the waiting period.

b Not applicable.

c WHYMPI: West Haven-Yale Multidimensional Pain Inventory [23].

d AQoL-8D: Assessment of Quality of Life—8 Dimension [24].

e CPAQ-D: Chronic Pain Acceptance Questionnaire, German version [25].

f GAD-7: Generalized Anxiety Disorder-7 [26].

g PHQ-9: Patient Health Questionnaire-9, German version [27].

#### Primary Outcomes

Primary outcomes include pain intensity, pain-related impairment, and health-related quality of life. To assess pain intensity and impairment, the German version of the WHYMPI will be used [23]. The WHYMPI is a comprehensive 52-item questionnaire comprising 12 subscales that assess the multidimensional impact of chronic pain on patients’ daily lives. It includes scales for pain severity, pain-related interference, perceived life control, and affective distress. The inventory also evaluates patients’ perceptions of significant others’ responses to their pain (eg, solicitous or negative behaviors) and the frequency of engagement in everyday activities, including a general activity level score. Responses are recorded on a 7-point Likert scale.

Health-related quality of life will be measured using the German version of the Assessment of Quality of Life—8 Dimension (AQoL-8D) instrument [24]. The AQoL-8D is a validated multiattribute utility measure consisting of 35 items that cover 8 key dimensions of quality of life: independent living, happiness, mental health, coping, relationships, self-worth, pain, and senses. Response options vary between 4-point and 6-point scales depending on the domain. The AQoL-8D yields a psychometric score that reflects overall health-related quality of life.

#### Secondary Outcomes

Secondary outcomes include physical and emotional functioning, patients’ global improvement, pain acceptance, anxiety, and depressive symptoms. To assess pain acceptance, the German version of the Chronic Pain Acceptance Questionnaire (CPAQ-D) will be used [25]. The CPAQ-D contains 20 items across 2 subscales: “Activity engagement” and “Pain willingness.” Responses are given on a 7-point scale, with higher scores indicating greater acceptance of chronic pain. Depressive symptoms will be assessed using the Patient Health Questionnaire-9 (PHQ-9) [27] and the Generalized Anxiety Disorder 7 (GAD-7) for anxiety symptoms [26]. The PHQ-9 includes 9 items that correspond to the *DSM-IV* (*Diagnostic and Statistical Manual of Mental Disorders, Fourth Edition*) criteria for depression and are rated on a 4-point scale (0=not at all to 3=nearly every day). Severity is categorized based on the total score (0‐4=minimal, 5‐9=mild, 10‐14=moderate, 15‐19=moderately severe, and ≥20=severe depression). The GAD-7 comprises 7 items rated on a 4-point scale and assesses the frequency of anxiety symptoms over the past 2 weeks. Severity categories include minimal, mild, moderate, and severe anxiety. Patients’ subjective global improvement and perceived functionality are additionally derived from specific subscales of the WHYMPI, including general activity level and affective distress, as indicators of change in physical and emotional functioning over the course of the intervention.

### Statistical Analysis

All statistical analyses will be conducted using the statistical software R (version 4.5.0; R Core Team). For the PP analysis, “full adherence” will be defined a priori. Participants must complete at least 9 out of the 12 intervention modules (≥75% completion rate) within the intended 12-week intervention period to be included in the PP dataset. Thresholds such as completing ≥75% of modules are commonly reported in digital intervention research to operationalize adherence criteria, although empirical justification varies across studies, and this threshold is considered consistent with existing eHealth adherence frameworks [28]. Participants who complete fewer than 9 modules or who finish all modules in an implausibly condensed time frame that violates the intended weekly structure will be excluded from the PP dataset. First, all primary and secondary outcomes as well as participant demographics and baseline characteristics will be analyzed descriptively (means, SDs, and frequencies), both for the total sample and stratified by the study group (EGpsych, EGcomp, and TAU). To examine intervention effects, mixed models for repeated measures will be used as the primary analytic approach. The mixed models for repeated measures allow the inclusion of all available outcome data and accommodate unequal assessment schedules between groups due to the 6-month waiting period in the TAU condition. The primary randomized comparison will focus on the matched assessment approximately 6 months after baseline, corresponding to the 3-month follow-up in the experimental groups (T2) and the end of the waiting period in the TAU group (TAU-T1). For each primary and secondary outcome, models will include fixed effects for group (EGpsych vs EGcomp vs TAU), time (baseline vs matched 6-mo assessment), and the group × time interaction. A participant-specific random intercept will be included to account for within-person correlation. The group × time interaction will be used to test differential change between conditions. Holm-corrected post hoc contrasts will be applied for pairwise comparisons where applicable. If model assumptions are violated, appropriate robust methods will be considered. Outcome data collected in the TAU group after participants have received access to CHRONIC (eg, TAU-T2 and TAU-T3) will not be included in the primary randomized comparison because TAU no longer represents a control condition at that stage. Post-waitlist TAU data will instead be analyzed separately and within-group (exploratory) to describe changes following delayed program access. Exploratory analyses within each study arm will be reported descriptively. However, comparisons across nonmatched time intervals will not be interpreted as randomized sensitivity analyses. When a significant main or interaction effect is detected, Holm-corrected post hoc tests will be applied to determine the specific time points and group differences, while controlling for the family-wise error rate.

All analyses will be conducted according to both the intention-to-treat and PP principles. The intention-to-treat approach includes all randomized participants in the groups to which they were assigned, regardless of adherence to the intervention, thereby maintaining the benefits of randomization and reflecting real-world applicability. In contrast, PP analyses include only those participants who fully adhered to the intervention protocol, offering insights into the intervention’s efficacy under ideal conditions. Participants must complete at least 9 out of 12 modules (≥75%) within the intended 12-week intervention period to be included in the dataset. Participants below this threshold will be excluded from PP analyses. Comparing both approaches helps assess the robustness and generalizability of findings. Subgroup analyses may be conducted to explore whether intervention effects differ across specific participant characteristics, such as baseline pain severity, age, or gender. These analyses can help identify moderators of treatment effects and thereby contribute to a more personalized understanding of intervention efficacy. Moderator analyses will be exploratory in nature and interpreted with caution due to limited power. Adherence outcomes, including the number of completed modules, log-in frequency, and intervention dropout, will be compared descriptively and inferentially between EGpsych and EGcomp to evaluate potential differences in engagement patterns. Missing data will be handled using multiple imputation, which estimates missing values based on the observed data and includes random variation across imputations. This approach reduces bias and increases statistical power compared to complete case analyses, as it uses all available data while accounting for uncertainty introduced by the missingness [29].

## Results

The study is conducted without external funding (internally financed). Recruitment began in January 2026 and is projected to end in July 2026. As of January 22, 2026, 18 participants had been enrolled. Primary end point data collection (6 mo after baseline) is expected to be completed in January 2027, with primary results planned for dissemination in 2027.

## Discussion

### Advantages and Challenges

Digital health interventions have gained considerable attention in recent years as effective tools for managing chronic pain. These interventions offer scalable and accessible treatment options that can complement traditional face-to-face care. Recent evidence supports their potential to reduce pain intensity, enhance physical functioning, and improve psychological outcomes in individuals living with chronic pain [30].

Pfeifer et al [20] conducted a systematic review and meta-analysis evaluating mobile app–based interventions for chronic pain. The study found significant improvements in pain intensity and physical function among users, highlighting the clinical relevance of mobile-supported self-management, especially in the long term. Similarly, Mecklenburg et al [9] reported positive effects of a multimodal 12-week digital care program combining sensor-guided exercises, education, and psychosocial support. Participants experienced reductions in pain and improvements in physical function, with a decreased intent to undergo surgery. This illustrates the potential of multicomponent digital programs to support effective long-term pain management. In addition to clinical efficacy, digital interventions can mitigate common barriers to accessing care. According to the International Association for the Study of Pain [31], digital psychosocial interventions allow for flexible, asynchronous delivery of care, which benefits individuals with limited mobility, caregiving responsibilities, or restricted access to specialized providers. Given its biopsychosocial foundation and multimodal structure, CHRONIC is particularly well-suited to address the complex needs of individuals with chronic pain in a scalable and accessible format.

Despite these advantages, challenges remain. Sustained engagement, digital literacy, and the seamless integration of these tools into existing health care systems require further development. Future studies should address long-term effects, cost-effectiveness, and personalization strategies to maximize benefits. In summary, digital health interventions represent a promising and evidence-based approach to chronic pain management. By leveraging technology to deliver psychological and behavioral interventions, they can improve patient outcomes, extend access to care, and support sustainable health care delivery models. Continued innovation and rigorous evaluation will be essential to unlocking their full potential. To our knowledge, this is the first study to systematically examine a combined psychotherapeutic and physiotherapeutic digital training program for individuals with chronic pain in Germany. Unlike existing programs that focus exclusively on either psychological or physical domains, this approach addresses both dimensions of chronic pain simultaneously—reflecting current biopsychosocial treatment recommendations. In summary, the findings of this RCT are expected to contribute to the development of integrated, scalable, and interdisciplinary care models in the digital health sector.

### Limitations

This study protocol outlines the planned design and methodology of an RCT evaluating the digital intervention CHRONIC. Several limitations should be considered in advance of implementation. First, the monocentric design may limit the generalizability of findings to broader populations or other clinical settings. Second, the digital format, while enhancing scalability and accessibility, may exclude individuals with limited internet access or low digital literacy, potentially introducing a selection bias. Third, all outcome measures rely on self-reported data, which may be affected by recall bias, social desirability, or inaccurate self-assessment. Fourth, participants are recruited via self-registration and online platforms, which may result in a self-selection bias favoring more motivated or health-literate individuals. Finally, although the trial compares 2 feedback modalities and a waitlist control, it does not include a nondigital active control group, which limits the ability to draw conclusions about the relative efficacy of digital versus traditional multimodal interventions. Another limitation is the absence of a face-to-face multimodal comparison arm, which would allow for a direct comparison between the digital intervention and traditional in-person care. Future studies should examine whether the effectiveness of CHRONIC is comparable to established multimodal face-to-face programs.

## Abbreviations

ACT: acceptance and commitment therapy
AQoL-8D: Assessment of Quality of Life–8 Dimension
CBT: cognitive behavioral therapy
DSM-IV: Diagnostic and Statistical Manual of Mental Disorders, Fourth Edition
EGcomp: experimental group with computer-generated feedback
EGpsych: experimental group with individualized psychological feedback
GAD-7: Generalized Anxiety Disorder-7
PHQ-9: Patient Health Questionnaire-9
PP: per-protocol
RCT: randomized controlled trial
TAU: treatment-as-usual
WHYMPI: West Haven-Yale Multidimensional Pain Inventory

## References

[1] Herausforderung Schmerz [Web page in German]. Deutsche Schmerzgesellschaft e.V..

[2] Zgierska AE, Edwards RR, Barrett B (2025). Mindfulness vs cognitive behavioral therapy for chronic low back pain treated with opioids: a randomized clinical trial. JAMA Netw Open.

[3] Deslauriers S, Roy JS, Bernatsky S (2021). The burden of waiting to access pain clinic services: perceptions and experiences of patients with rheumatic conditions. BMC Health Serv Res.

[4] Lin J, Paganini S, Sander L (2017). An internet-based intervention for chronic pain. Dtsch Arztebl Int.

[5] Trompetter HR, Bohlmeijer ET, Veehof MM, Schreurs KMG (2015). Internet-based guided self-help intervention for chronic pain based on acceptance and commitment therapy: a randomized controlled trial. J Behav Med.

[6] Dear BF, Gandy M, Karin E (2018). The pain course: 12- and 24-month outcomes from a randomized controlled trial of an internet-delivered pain management program provided with different levels of clinician support. J Pain.

[7] Gatchel RJ, McGeary DD, McGeary CA, Lippe B (2014). Interdisciplinary chronic pain management: past, present, and future. Am Psychol.

[8] Kamper SJ, Apeldoorn AT, Chiarotto A (2015). Multidisciplinary biopsychosocial rehabilitation for chronic low back pain [Article in Spanish]. Cochrane Database Syst Rev.

[9] Mecklenburg G, Smittenaar P, Erhart-Hledik JC, Perez DA, Hunter S (2018). Effects of a 12-week digital care program for chronic knee pain on pain, mobility, and surgery risk: randomized controlled trial. J Med Internet Res.

[10] Toelle TR, Utpadel-Fischler DA, Haas KK, Priebe JA (2019). App-based multidisciplinary back pain treatment versus combined physiotherapy plus online education: a randomized controlled trial. NPJ Digit Med.

[11] Hayes SC, Strosahl KD, Wilson KG (1999). Acceptance and Commitment Therapy: An Experiential Approach to Behavior Change.

[12] Segal ZV, Williams JMG, Teasdale JD (2013). Mindfulness-Based Cognitive Therapy for Depression.

[13] Hylands-White N, Duarte RV, Raphael JH (2017). An overview of treatment approaches for chronic pain management. Rheumatol Int.

[14] Skelly AC, Chou R, Dettori JR (2020). Noninvasive nonpharmacological treatment for chronic pain: a systematic review update (comparative effectiveness review no227). https://effectivehealthcare.ahrq.gov/sites/default/files/related_files/noninvasive-nonpharm-pain-update.pdf.

[15] Terminology. International Association for the Study of Pain (IASP).

[16] Apkarian AV, Baliki MN, Geha PY (2009). Towards a theory of chronic pain. Prog Neurobiol.

[17] Zinboonyahgoon N, Luansritisakul C, Eiamtanasate S (2021). Comparing the ICD-11 chronic pain classification with ICD-10: how can the new coding system make chronic pain visible? A study in a tertiary care pain clinic setting. Pain.

[18] Treede RD, Rief W, Barke A (2015). A classification of chronic pain for ICD-11. Pain.

[19] Flores MP, de Castro A, Nascimento J dos S (2012). Topical analgesics [Article in Portuguese]. Braz J Anesthesiol.

[20] Pfeifer AC, Uddin R, Schröder-Pfeifer P, Holl F, Swoboda W, Schiltenwolf M (2020). Mobile application-based interventions for chronic pain patients: a systematic review and meta-analysis of effectiveness. J Clin Med.

[21] Shetty A, Delanerolle G, Zeng Y (2022). A systematic review and meta-analysis of digital application use in clinical research in pain medicine. Front Digit Health.

[22] DiGA-verzeichnis [Web page in German]. Bundesinstitut für Arzneimittel und Medizinprodukte.

[23] Flor H, Rudy TE, Birbaumer N, Streit B, Schugens MM (1990). Zur Anwendbarkeit des West Haven-Yale Multidimensional Pain Inventory im deutschen Sprachraum [Article in German]. Schmerz.

[24] Maxwell A, Özmen M, Iezzi A, Richardson JR (2016). Deriving population norms for the AQoL-6D and AQoL-8D multi-attribute utility instruments from web-based data. Qual Life Res.

[25] Nilges P, Köster B, Schmidt CO (2011). CPAQ-D - Chronic Pain Acceptance Questionnaire - Deutsche Fassung [Article in German].

[26] Löwe B, Decker O, Müller S (2008). Validation and standardization of the Generalized Anxiety Disorder Screener (GAD-7) in the general population. Med Care.

[27] Löwe B, Spitzer RL, Zipfel S, Herzog W (2003). PHQ-D. Gesundheitsfragebogen für Patienten: 2. Auflage, Pfizer GmbH, Karlsruhe, 2002, kostenlos [Article in German]. Z Med Psychol.

[28] Sieverink F, Kelders SM, Gemert-Pijnen JEWC van (2017). Clarifying the concept of adherence to eHealth technology: systematic review on when usage becomes adherence. J Med Internet Res.

[29] Jakobsen JC, Gluud C, Wetterslev J, Winkel P (2017). When and how should multiple imputation be used for handling missing data in randomised clinical trials - a practical guide with flowcharts. BMC Med Res Methodol.

[30] Lee J, Mowat R, Blamires J, Foster M (2025). Recent advances in non-invasive digital nursing technologies for chronic pain assessment and management: an integrative review. J Adv Nurs.

[31] (2022). Digital health psychosocial interventions for chronic pain. International Association for the Study of Pain (IASP).

